# Challenges in diagnosis of genital ulcers: a genital leishmaniasis case series

**DOI:** 10.1590/0037-8682-0772-2020

**Published:** 2021-03-22

**Authors:** Marina Dias de Souza, Aloísio Falqueto, Gabriela Lopes de Morais, Amanda da Silva Salomão, Fernanda Daher Pereira

**Affiliations:** 1 Universidade Federal do Espírito Santo, Departamento de Doenças Infecciosas, Vitória, ES, Brasil.

**Keywords:** Leishmaniasis, Mucocutaneous, Genital ulcers

## Abstract

Leishmaniasis is a tropical infectious disease caused by *Leishmania spp. protozoa* and is transmitted by insects from the Phlebotominae subfamily. It can manifest as cutaneous leishmaniasis, a painless ulcer that can develop into a more serious systemic affliction as the protozoa spreads lymphatically or hematogenously, depending on the host's immunity. In this case series, the authors present a rare form of genital mucocutaneous leishmaniasis, with consideration of epidemiologic characteristics, clinical presentation, differential diagnosis, and treatments offered.

## INTRODUCTION

Leishmaniasis is an endemic zoonosis in 88 countries caused by an intracellular *Leishmania* parasite transmitted by sand flies^1^. The most common hosts are dogs, rodents, and foxes; humans are accidental hosts^2^. Cutaneous leishmaniasis (CL) is the most common clinical presentation, and clinical features depend on the species’ virulence and host immune responses^3^. It presents in exposed areas of the body as a rounded, non-painful erythematous ulcer with thick borders, raised edges, and a granulated red peripheral area^4^. Seven species were identified in Brazil as causing cutaneous diseases^5^. In Espírito Santo, only the most widespread species was found: *Leishmania (Viannia) braziliensis,* which is responsible for mucosal disease^6^.

Pleomorphisms of CL include the lesion characteristics, affected regions^2^, and genital manifestations. The cause of these atypical manifestations are unclear, although immunosuppression is an important factor in some cases^2^ ^,^ ^7^. To avoid deformations or functional sequelae, early diagnosis is essential to begin treatment, since genital ulcers are common and could have multiple etiologies, and there are very few publications describing genital leishmaniasis. To our knowledge, this is the first case report series in Brazil to expand the conditions for differential diagnosis in an area endemic for leishmaniasis. 

## CASE REPORT

**FIGURE 1:**
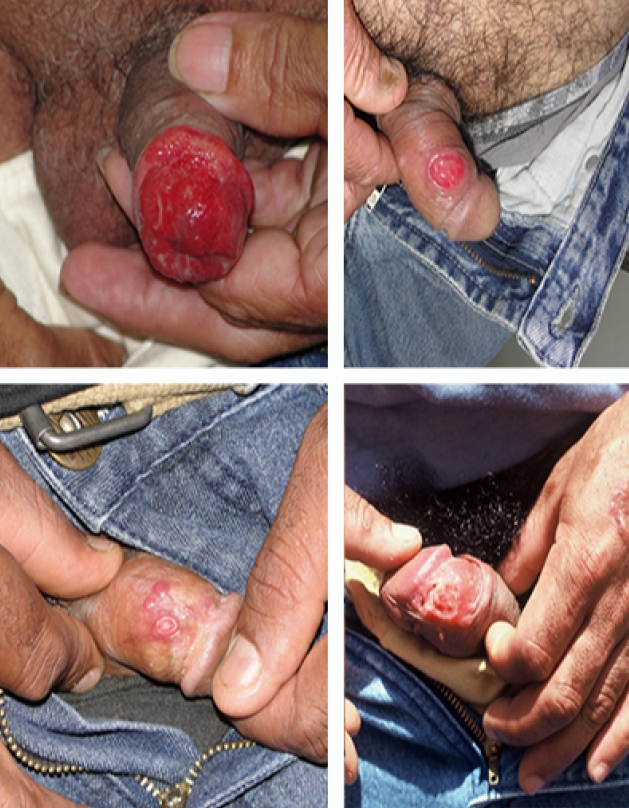
Photographs of genital lesions from cases 1 to 4, respectively.

The first patient presented with a small pruritic lesion on the penis, which progressed for 20 years with ineffective use of benzathine penicillin. He was treated for CL for three years before the wound appearance. An excisional biopsy of the lesion showed necrotizing non-caseous epithelioid granulomatous inflammation, the presence of vasculitis, and was negative for tuberculosis and fungal results. A rough and infiltrated uvula, slight roughness in the bilateral nasal septum, and an ulcerated, hyperemic, and infiltrated lesion surrounding the penis glans are shown in Figure 1. The Montenegro skin test results were positive (15 mm). The lesions were treated with three cycles of 10-20 mg/kg per day of pentavalent antimonials for 20 days, which led to complete remission of symptoms and satisfactory healing of the lesions. 

The second patient had a six-month-old ulcerated lesion on the foreskin, 1.5 cm in diameter, with raised erythematous borders and a clean, slightly depressible, painless center. The patient was treated unsuccessfully with benzathine penicillin. Pentavalent antimonials had been used two years prior, which suggested reactivation. Examination of the penile lesion revealed an amastigote form of the parasite, which was successfully treated with pentavalent antimonials.

The third patient underwent previous treatment for CL in the sternal furcula, face, and pinna with a good clinical response. One year after his first treatment, the patient returned complaining of a non-itching and painless lesion on the penis that had started two months prior. Serology for hepatitis, syphilis, and human immunodeficiency virus (HIV) were non-reactive. There was a penile ulcer in the balanoprepucial groove (0.6 cm) and two papular proximal lesions (0.5 cm) on the penis dorsal surface. Direct examination of the lesions found intra- and extra-cellular forms of the parasite, and two cycles of pentavalent antimonials were prescribed.

The fourth patient presented a vegetating lesion on the lower lip, an ulcerated lesion affecting the nasogastric groove and wing of the nose (external and internal faces), and an ulcerated lesion on the left side. Approximately two months after their appearance, the patient reported three ulcerated lesions on the penis: one on the balanoprepucial groove and two on the glans. The Montenegro skin test showed a 7 mm result, and a biopsy of the penile lesion with histology suggested leishmaniasis. Pentavalent antimoniate treatment was not initially successful. All injuries healed after three additional treatment cycles.

**FIGURE 2:**
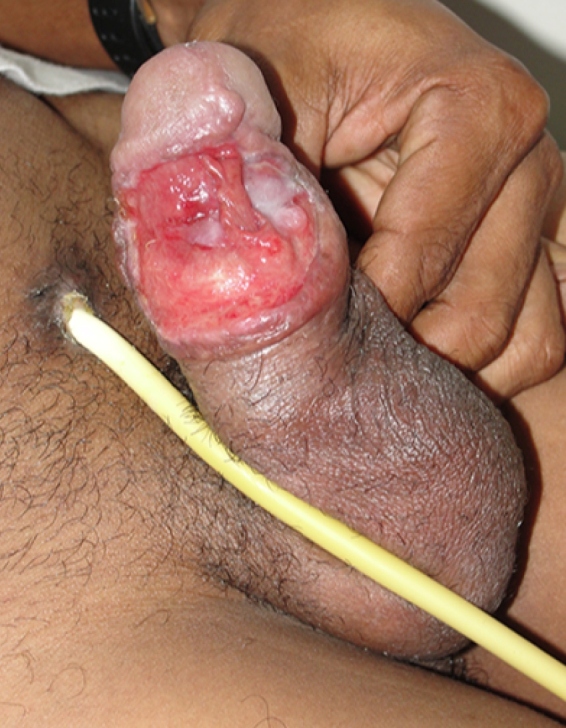
Photograph of genital ulcer with exposure of corpora cavernosa and the distal urethra.

The fifth patient presented with an extensive painful ulcer with elevated and well-defined edges, an erythematous and granular surface affecting the body of the penis and glans, and exposure of the corpora cavernosa and distal urethra (Figure 2). Eleven years prior, he had been treated for CL with pentavalent antimonials. A cystostomy was performed, and histopathological results suggested syphilis, although the Venereal Disease Research Laboratory test was non-reactive in the peripheral blood. The patient was diagnosed with HIV infection. In addition to the genital lesion, the patient had cervical lymph node enlargement and hepatomegaly. Direct examination of the lesion confirmed leishmaniasis. After discharge, the patient returned complaining of two bloody areas with granular tissue. He underwent two more cycles of antimonials, with complete remission of symptoms, satisfactory healing of the lesion, and removal of the cystostomy.

**FIGURE 3:**
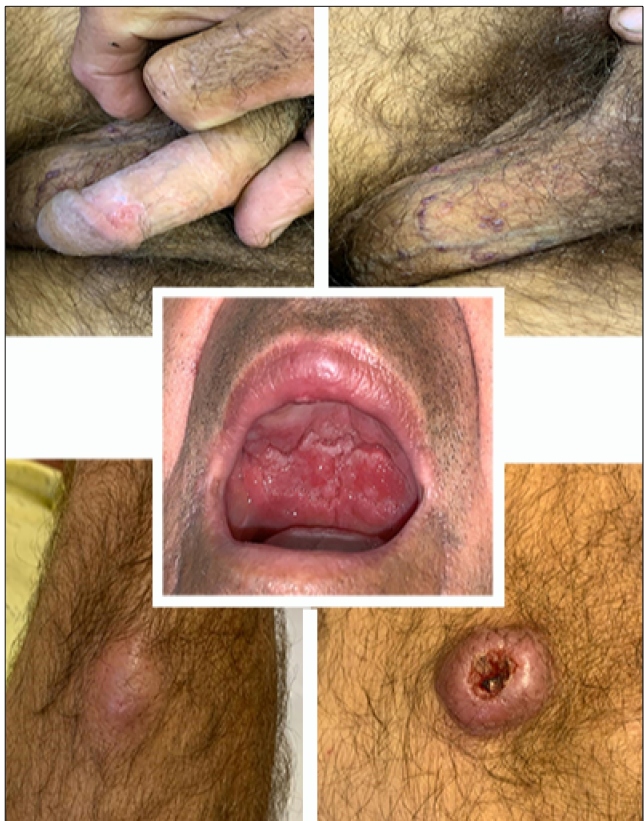
Photograph of disseminated cutaneous-mucous leishmaniasis lesions.

The sixth patient presented with nodular lesions on the forearm and knee without specific treatment. After two months, the patient developed an ulcer on the granular bottom lip mucosa with a progressive and painless size increase. It was associated with an extensive ulcerated lesion on the soft palate (Figure 3) and upper gingival ridge, with infiltration of the uvula and amygdala. There were multiple papules in the genital region, with a granular surface, a wine-red color, and a central crust at the scrotum and base of the penis. A well-defined ulcer with high edges, a deep crusty center, and a small amount of secretion was found on the left knee. Direct examination revealed the amastigote form of the protozoan. The patient was diagnosed with disseminated cutaneous mucous leishmaniasis and had a good response to one cycle of pentavalent antimonials.

The seventh patient had an ulcerated erythematous lesion with well-defined borders on his right forearm, which progressively increased in size. Similar lesions appeared on the left forearm, face, and temporal region bilaterally and, lastly, on the penis. Biopsy of the forearm lesion showed superficial and deep dermatitis with areas of granuloma and intense mononuclear infiltrate with lymphocytes, plasmocytes, and histiocytes. After one month, bleeding ulcers on the nasal mucosa appeared. Direct examination confirmed the diagnosis of disseminated CL. Treatment with pentavalent antimonials led to complete regression and scarring.

## DISCUSSION

American tegumentary leishmaniasis (ATL) is very common in Brazil, with wide territorial expansion and focal transmission. Globally, there are 0.7-1.3 million cases among 85 countries^2^ ^,^ ^5^ ^,^ ^8^. Clinical features of CL consist of ulcers exclusively on the skin, and reappearance of cutaneous and mucosal lesions is possible due to hematogenous or lymphatic spread of the parasite. The rarest manifestation of ATL is diffuse CL, in which lesions are not near the bite; localized CL has wounds near the bite; mucocutaneous leishmaniasis may manifest either way^9^. Genital lesions are comparable to mucous lesions and are caused by direct inoculation by sand fly bites or from hematogenous spread^10^.

In most cases, cutaneous lesions appear in exposed body areas, with raised edges and a granular surface, which are painless, slow growing, and eventually cause significant devitalization of adjacent tissue. Since little is known about leishmaniasis located in the genital area, these cases reveal the importance of physically examining mucosal and skin lesions and obtaining detailed epidemiological and clinical histories. Genital lesions may appear close to sand fly bites or result from blood dissemination of the infection^1^ ^,^ ^11^. Detecting the disseminated form allows the physician to identify chronic genital ulcers that do not respond to conventional treatments.

The Montenegro skin test is an indirect diagnostic method performed by intradermal antigen injection to measure the sensitization of the host against the parasite. Patients with long-term diseases, especially mucosal, show exacerbated responses; in these cases, the positivity index is nearly 100%. A non-reactive Montenegro skin test in patients with mucosal lesions has a high negative predictive value, and differential diagnoses should be considered^7^.

Direct visual confirmation of the parasite is required for diagnosis and has a 90% positive predictive rate, surpassing histopathological accuracy. However, experienced physicians are required. Histopathological features depend on the host’s immune characteristics and the length of disease evolution. Biopsy is essential, despite its low sensitivity, since CL ulcers provide differential diagnosis of deep mycoses, cutaneous lymphomas, basal cell carcinoma, cutaneous anthrax, leprosy, syphilis, and other conditions^2^.

All patients reported for the disseminated form of the disease are male, between 25-65 years old, living at rural sites, and half were smokers and consumed alcohol. Genital lesions were followed by disseminated or recurrent forms, which ranged from to 1-11 years after the first manifestation of cutaneous disease. In all cases, epidemiologic histories were from areas endemic for cutaneous mucous leishmaniasis.

Comorbidities, especially HIV-related immunosuppression, indicate greater severity and prevalence of atypical lesions, including the genital form^7^. The patient suffering from this condition presented the most aggressive and incapacitating symptoms, with exposure of the urethra and the need for cystostomy, suggesting the host’s immune system directly influenced the severity of clinical presentation.

CL can manifest in several ways, leading to possible delays in diagnosis and treatment. The patient's epidemiological background is essential for differential diagnosis of cutaneous mucous ulcers, along with a direct examination performed by an experienced professional. Montenegro skin tests can corroborate, but diagnosis is confirmed with visualization of the parasite, but they are not currently available in Brazil. Treatment with pentavalent antimonials remains the most effective treatment for all cases, even atypical forms.

Genital leishmaniasis should be considered in differential diagnosis for genital ulcers, especially in male patients from areas prevalent with the disease vector or in patients previously treated for cutaneous or disseminated leishmaniasis.
